# Alterations in Small Intestine and Liver Morphology, Immunolocalization of Leptin, Ghrelin and Nesfatin-1 as Well as Immunoexpression of Tight Junction Proteins in Intestinal Mucosa after Gastrectomy in Rat Model

**DOI:** 10.3390/jcm10020272

**Published:** 2021-01-13

**Authors:** Iwona Puzio, Siemowit Muszyński, Piotr Dobrowolski, Małgorzata Kapica, Marta Pawłowska-Olszewska, Janine Donaldson, Ewa Tomaszewska

**Affiliations:** 1 Department of Animal Physiology, Faculty of Veterinary Medicine, University of Life Sciences in Lublin, Akademicka St. 12, 20-950 Lublin, Poland; malgorzata.kapica@up.lublin.pl (M.K.); martapaw@autograf.pl (M.P.-O.); ewaRST@interia.pl (E.T.); 2 Department of Biophysics, Faculty of Environmental Biology, University of Life Sciences in Lublin, Akademicka St. 13, 20-950 Lublin, Poland; 3 Department of Functional Anatomy and Cytobiology, Faculty of Biology and Biotechnology, Maria Curie-Sklodowska University, Akademicka St. 19, 20-033 Lublin, Poland; piotr.dobrowolski@umcs.lublin.pl; 4 School of Physiology, Faculty of Health Sciences, University of the Witwatersrand, 7 York Road, Parktown, Johannesburg 2193, South Africa; janine.donaldson@wits.ac.za

**Keywords:** gastrectomy, gut hormones, tight junction, stomach, enteroendocrine cells, small intestine

## Abstract

The stomach is responsible for the processing of nutrients as well as for the secretion of various hormones which are involved in many activities throughout the gastrointestinal tract. Experimental adult male Wistar rats (*n* = 6) underwent a modified gastrectomy, while control rats (*n* = 6) were sham-operated. After six weeks, changes in small intestine (including histomorphometrical parameters of the enteric nervous plexuses) and liver morphology, immunolocalization of leptin, ghrelin and nesfatin-1 as well as proteins forming adherens and tight junctions (E-cadherin, zonula occludens-1, occludin, marvelD3) in intestinal mucosa were evaluated. A number of effects on small intestine morphology, enteric nervous system ganglia, hormones and proteins expression were found, showing intestinal enteroplasticity and neuroplasticity associated with changes in gastrointestinal tract condition. The functional changes in intestinal mucosa and the enteric nervous system could be responsible for the altered intestinal barrier and hormonal responses following gastrectomy. The results suggest that more complicated regulatory mechanisms than that of compensatory mucosal hypertrophy alone are involved.

## 1. Introduction

The stomach is responsible for the mechanical and enzymatic processing of nutrients and participates in the regulation of acid secretion, nutrient assimilation, appetite, metabolism and energy homeostasis of the body as well as in the secretion of various hormones. Surgical treatment, which includes total gastrectomy, remains the basic method of treatment for patients with gastric cancer. However, after total gastrectomy, the patient requires further systematic care, including reconstruction of the gastrointestinal tract (GIT). Although the literature provides more than 50 methods of reconstruction of the gastrointestinal tract, none of the methods improve the life quality, which decreases rapidly with surgery [1]. Total gastrectomy irreversibly disrupts the physiology of the digestive system, leading to the loss of capacitive function and the disappearance of the secretion of digestive enzymes and hormones. Many patients that undergo gastrectomy experience long-lasting gastrointestinal symptoms and complications following the procedure, which include mechanical, metabolic, deficient, circulatory, psychosocial and economic complications [2]. Among them are anemia, as a result of iron and vitamin deficiencies, reflux, diarrhea, steatorrhea, increased bowel movements and loss of appetite, all of which can result in reduced overall well-being, malnutrition and weight loss [3,4]. While the exact etiology of these symptoms is still unclear, numerous studies indicate that the mechanisms responsible for the complications following surgery involve independent alterations in gastrointestinal motility [5], vagal innervation [6], hormonal [7] signaling pathways, bile acids [8] and intestinal microbiota [9]. 

Enteroplasticity following gastrectomy has been extensively studied; however, much less is known regarding neuroplasticity as the reorganization of enteric innervations [10,11,12,13,14]. This adaptation relates to changes in the external and internal environment, and except functional changes (the number and transmission of synaptic connections, modification of intracellular signaling cascades and regulation of gene expression), it includes alterations in the synthesis and release of neurotransmitters. Studies in animal models have showed that many various factors trigger a response of enteric neurons, expressed as a change in their neurochemical characteristics [15,16,17].

In order to improve the quality of life for a growing group of patients undergoing gastrectomy, it is necessary to understand the possible range of complications resulting from gastrectomy and the possibilities of their treatment or mitigation. To the best of our knowledge, studies about the possible effects of total gastrectomy on the expression of proteins forming intercellular junctions in the small intestine are missing. In addition, the expression of most stomach-derived peptides within the GIT following gastrectomy has not been studied. As the absence of stomach also reveals liver dysfunction, there is a need to investigate the impact of gastrectomy on liver structure.

To provide an adequate explanation for what occurs after gastrectomy, the aim of the presented study was to examine its effects on the jejunal, duodenal and liver morphology; the immunolocalization of nesfatin-1, ghrelin, leptin and proteins forming adherens and tight junctions (E-cadherin, zonula occludens-1, occludin and marvelD3) in intestinal mucosa as well as neuroplasticity of the enteric nervous system in a rat model of total gastrectomy.

## 2. Experimental Section

### 2.1. Experimental Design

All procedures using animals that were carried out were approved by the Local Ethics Committee for Animal Experiments, University of Life Sciences in Lublin, Poland (No. 64/2012) and were performed according to the Guiding Principles for Research Involving Animals.

**Figure 1:**
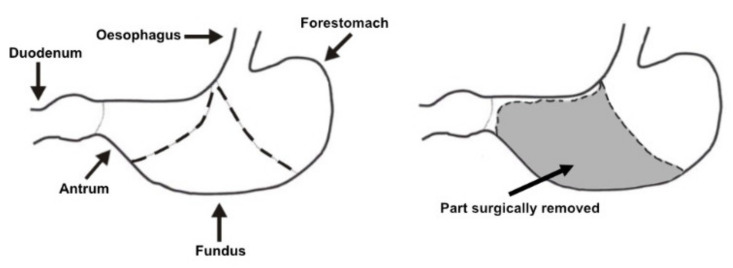
Schematic presentation of the performed modified gastrectomy.

The experiment was carried out on twelve adult male Wistar rats with a body weight of approximately 220–240 g. After a 7-day adaptation period to the experimental conditions of the vivarium (temperature 22 °C ± 2%, humidity 55 ± 10%, and a 12-h day/night cycle), the rats were randomly divided into control (CONT, *n* = 6) and experimental (GASTR, *n* = 6) groups. CONT rats underwent a sham operation, which involved a midline incision of the abdominal wall, gentle reposition of viscera, and the incision was then stitched closed. GASTR rats underwent a modified gastrectomy (Figure 1), during which all glandular parts of the rat stomach (fundus and antrum) were removed, and a connection between the remaining rumen (non-glandular part of the rat stomach) and the duodenum (end-to-end) was then established, with care taken to preserve the vagus nerve [18,19,20,21]. Rats were fasted for 12 h prior to surgery. General anesthesia was used for all surgical procedures, with ketamine (15 mg/kg b.w. *i.m.*) and xylazine hydrochloride (35 mg/kg b.w. *i.m.*). After surgery, rats were administered amoxicillin for 3 days (30 mg/rat *i.m.*) and were housed under the controlled conditions of the vivarium for a period of 6 weeks. Rats showed proper behavior and did not show symptoms (bleeding, anastomotic failure, infection, reflux, diarrhea, increased bowel movement and decreased feed intake), which can indicate post-operative complication. Rats were fed ad libitum a standard laboratory rodent diet, formulated to meet minimal nutritional requirements specified in AIN-93M [22] and had free access to water. The diet contained 160 g protein, 28 g fat, 50 g crude fiber and 70 g crude ash in 1 kg of feed, with metabolizable energy of 11 MJ/1 kg feed dry mass. At the end of the 6th week of the experimental period, the rats were fasted overnight (12 h) and then anesthetized at the same time in the morning next day. Duodenum, jejunum and liver samples were then collected for further analysis. In the present study, no-operated group of rats was excluded in accordance with the “3Rs” principle and the Ethics Committee recommendation in order to avoid unnecessary use of experimental animals.

### 2.2. Tissue Collection and Histomorphometry Analysis

Two 10-mm long segments of the small intestine, from the duodenum (20 mm distal to the pylorus) and from the jejunum (50% of the total intestinal length of the jejunum), as well as 0.5-cm^3^ samples from the right lobe of the liver were taken from each rat. Intestine samples were opened along the mesentery and placed flat, without stretching, in such a way so as to avoid contact of the mucosa with the histopathological cassette walls [23]. Tissues were fixed in 4% buffered formaldehyde (pH 7.0) for 24 h, dehydrated in a graded series of ethanol, cleared with a nonpolar solvent and then embedded in paraffin. Then, 4-μm thick cross-sections were cut with a microtome (Microm HM 360, Walldorf, Germany), then placed on one microscopic slide (Polysine™, Menzel Glaser, Braunschweig, Germany) and stained with Masson’s trichrome (MT) and PicroSirius red (PSR) [24]. Stained slides were observed in brightfield (Masson’s trichrome) and polarized light (PicroSirius red) using an AXIOVERT 200 M confocal microscope (Carl Zeiss, Jena, Germany) as well as a CX43 (Olympus, Tokyo, Japan) microscope. Collected microscopic images were examined blindly by an associate who was not aware of the treatment using the following graphical analysis software: Olympus cellSens (Olympus, Tokyo, Japan) and ImageJ [25].

Microscopic observations allowed to identify and assess the structure and morphology of the liver tissue samples. For the intestine samples, the following morphometric parameters were analyzed: the thickness of the mucosa, submucosa, myenteron and villar epithelium; enterocyte number; the number of villi, active and inactive crypts; villus length and thickness; crypt depth and width; area of the small intestine absorptive surface [23,26]. The measurements of each variable were made on three separate tissue sections, on at least ten different areas of each section. The measurements were then averaged and expressed as the mean value of calculated parameters for each rat. Other analyses including intestine and liver tissues were performed in accordance with an earlier description [23]. PSR staining was used to differentiate collagen fibers in examined tissue samples, where the type I (thicker, mature) collagen fibers are orange or red and the type III (thinner, immature) are green in polarized light [24].

### 2.3. Immunohistochemistry

Immunohistochemical staining was performed on the remaining tissue slices after deparaffinization in xylene and rehydration with decreased concentrations of ethanol and distilled water. Heat-induced epitope retrieval was performed using a Rapid Cook pressure cooker (Morphy Richards, Swinton, UK) in sodium citrate buffer (10 mM sodium citrate, 0.05% Tween 20, pH 6.0), according to the protocols provided by the producers of the antibodies. Endogenous peroxidase activity was blocked subsequently with a 3% solution of hydrogen peroxide in deionized water for 5 min. After blocking for 30 min in normal serum, sections were incubated with the first antibodies overnight at 4 °C. All primary antibodies were rat-specific: rabbit polyclonal anti-Ki67 antibody (ab15580, Abcam, Cambridge, UK, dilution 1:50); rabbit polyclonal anti-neurofilament heavy subunits neuronal marker to localize Meissner and Auerbach plexuses (ab8153, Abcam, Cambridge, UK, dilution 1:200); rabbit polyclonal anti-nesfatin-1 antibody (H-003-22, Phoenix Pharmaceuticals, Burlingame, CA, USA, dilution 1:2000); mouse monoclonal anti-E cadherin antibody to mark adherence-type cellular junctions in the small intestine epithelium (ab231303, Abcam, Cambridge, UK, dilution 1:500); three types of antibodies were used to mark tight junctions—rabbit polyclonal anti-ZO-1 (zonula occludens 1) antibody (orb313868, Biorbyt, St. Louis, MO, USA, dilution 1:500), rabbit polyclonal MarvelD3 antibody (PA5-42629, Invitrogen, Thermo Fisher Scientific, Waltham, MA, USA, dilution 1:100) and rabbit polyclonal anti-occludin antibody (ab222691; Abcam, Cambridge, UK, dilution 1:100). Two “hunger-related” enteroendocrine hormones were detected with the use of rabbit polyclonal anti-ghrelin antibody (ab217011; Abcam, Cambridge, UK, dilution 1:50) and rabbit polyclonal anti-leptin antibody (ab16227; Leptin; Abcam, Cambridge, UK, dilution 1:50); rabbit polyclonal anti-VIP antibody was used to localize and identify the expression of vasoactive intestinal peptide (VIP) (ab22736; Abcam, Cambridge, UK, dilution 1:400). The sections were then incubated for 30 min with the appropriate second antibodies (peroxidase-conjugated goat anti-rabbit, #611-1302, Rockland Immunochemicals, Inc. Limerick, IL, USA, dilution 1:500, or peroxidase-conjugated goat anti-rabbit, ab6721, Abcam, Cambridge, UK, dilution 1:200). Negative control sections for each antibody were obtained by identical immunohistochemical staining excluding the primary antibody (Figure S1). The sections were then developed in 3,3′-diaminobenzidine tetrahydrochloride (DAB D5905; Sigma-Aldrich, St. Louis, MO, USA) or 3,3′-diaminobenzidine tetrahydrochloride with a metal enhancer (SIGMAFAST™ DAB D0426; Sigma-Aldrich, St. Louis, MO, USA), both used as chromogens for 15 min at room temperature. Counterstaining was performed with Mayer’s hematoxylin (MHS32-1L; Sigma-Aldrich, St. Louis, MO, USA) or Nuclear Fast Red counterstain (H-24-2; Vector Laboratories Inc., Burlingame, CA, USA), respectively.

Microscopic images were subjected to further analysis. For Ki-67, the proliferating cell index (the percent of proliferating cells in relation to all the gland cells) and the number of proliferating cells per 0.01 mm^2^ of the gland surface were determined. For neurofilament detection, the cross-sectional area of the nerve ganglion, the sphericity, perimeter, the minimal and mean diameters of the ganglion and the mean Feret diameter (the distance between the two tangential lines restricting the object perpendicular to that direction) were determined using ImageJ software [25].

The intensity of immunoreaction (brown color or gray, depending on staining) was measured by the quantitative comparison of mean pixel intensity values in the photomicrographs converted into 8-bit grayscale images. The scale was from 0 (white pixel) to 255 (black pixel); the lower the pixel value, the higher the intensity of immunohistochemical reaction [27]. The intensity of the immunoreaction in each of the analyzed digital images was measured in 10 randomly selected areas of the positive signal. The analyses were done blindly by an associate who was not aware of the treatment using ImageJ software [25].

### 2.4. Statistical Analysis

All statistical procedures were conducted using Statistica 13 software (TIBCO Software Inc., Palo Alto, CA, USA). Normal distribution of data was examined using the Shapiro–Wilk test and equality of variance was tested using Levene’s test. A comparison between normally distributed variables with equal variances was carried out using the two-tailed Student’s *t*-test or a *t*-test with Welch’s correction when normally distributed data lacked equal variances. When there was a lack of normal distribution, a non-parametric Mann–Whitney U analysis was used to test the differences between means. For all tests, a *p*-value of less than 0.05 was considered statistically significant. The results presented in the tables and in Figure 1 (body mass gain) are expressed as mean ± standard deviation (SD), whereas other graphs present results as mean ± standard error (SE). The results included in Supplementary Materials Table S1 present mean ± SE, median (with Q1 and Q3 quartile) and the exact *p*-value for all data and the effect size for all significant comparisons estimated with Cohen’s *d* for parametric comparisons and Cohen’s *r* for non-parametric comparisons.

## 3. Results

### 3.1. Morphology

**Figure 2:**
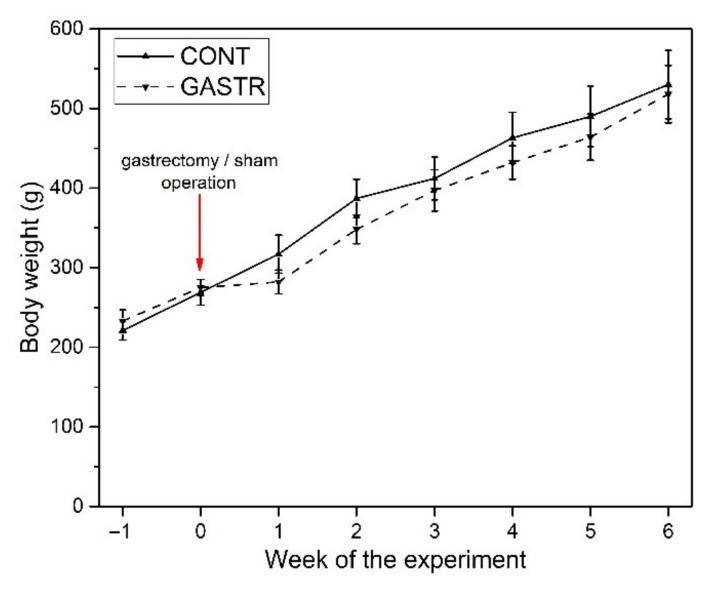
Changes in body weight of male Wistar rats in the CONT (sham-operated) and GASTR (subjected to gastrectomy) groups.

The initial and final body weights of the rats did not differ significantly between groups (Figure 2). In the first week after the gastrectomy, the inhibition of weight gain was observed, but it was also not statistically significant compared to the sham-operated (control) group (Figure 2).

**Figure 3:**
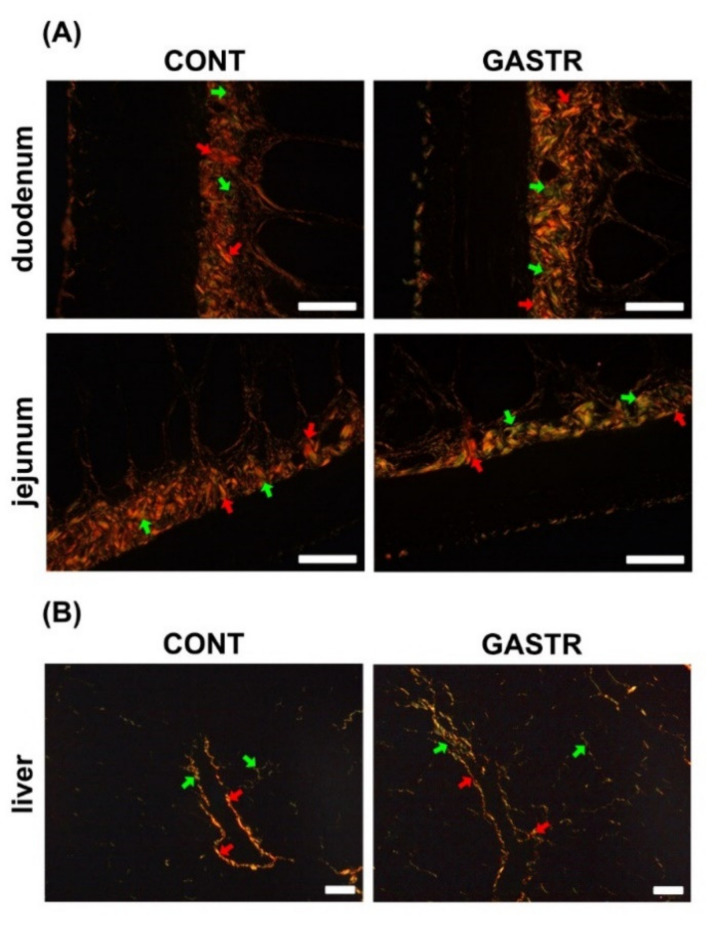
(**A**) Representative photomicrographs of PicroSirius red (PSR)-stained sections of the duodenum and jejunum. Mature, thick collagen fibers are red-orange, and immature, thin collagen fibers are green. Intestine sections from the CONT group, irrespective of the analyzed fragment, contained thick and thin fibers, with a predominance of thick fibers (red, mature). An increase in immature fibers (thin and green) was observed after gastrectomy. (**B**) Representative photomicrographs of PSR-stained sections of the liver. Liver sections from the CONT group contained thick and thin fibers, with a predominance of thick fibers (red, mature). An increase in immature fibers (thin and green) was observed in the gastrectomy-treated group. Red arrow indicates mature collagen; green arrow indicates immature collagen. All the scale bars represent 50 μm.

**Figure 4:**
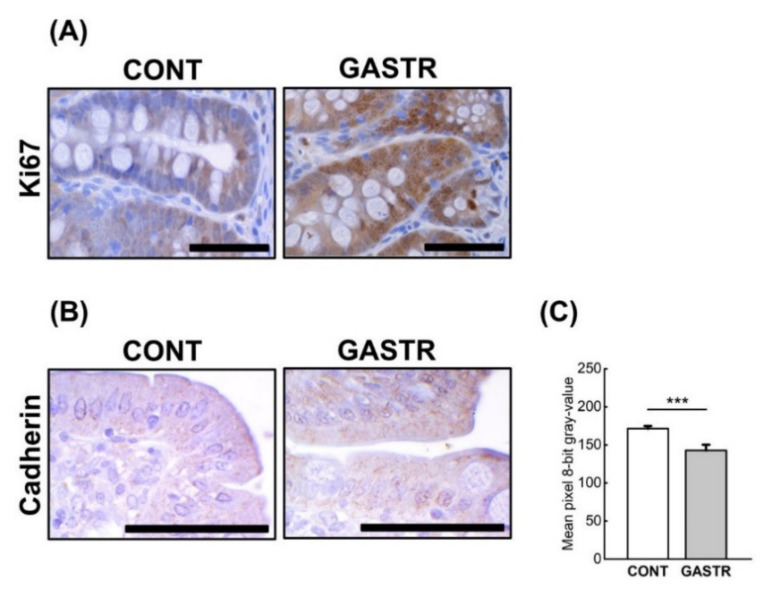
(**A**) Representative photomicrographs of the immunohistochemical reactions for Ki-67 in the jejunum. (**B**) Representative pictures of the immunohistochemical reactions for cadherin in the sections of the jejunum. Sections were developed in 3,3′-diaminobenzidine tetrahydrochloride (DAB); counterstaining was performed with Mayer’s hematoxylin. All the scale bars represent 50 μm. (**C**) The intensity of expression of cadherin in the jejunum, measured by the quantitative assessment of mean pixel intensity values in the photomicrographs converted to 8-bit grayscale images. The scale was from 0 (white pixel) to 255 (black pixel); the lower the pixel value, the higher the intensity of immunohistochemical reaction. Graph shows mean ± standard error. Significance was established using a two-tailed Student’s *t*-test (normally distributed data), Welch’s test (normally distributed data with unequal variances) or the Mann–Whitney test (for pairwise comparisons with at least one non-normally distributed dataset); *** *p* < 0.001.

No macroscopic changes indicating an anastomotic failure or infection were found during dissection. In the gastrectomy-treated group, there was a decrease in the thickness of the submucosa and the number of active crypts, while a significant increase in the content of immature collagen in the duodenum was observed (Table 1 and Figure 3A). In the jejunum, significant increases in the thicknesses of the myenteron, submucosa and mucosa were observed after gastrectomy (Table 1). Moreover, in the gastrectomy-treated group, an increase in the immature collagen content, Ki index and number of proliferating cells in the jejunum was noted (Table 1 and Figure 3A and Figure 4A), while the number of enterocytes was decreased in the GASTR group. The number of Meissner ganglia in the duodenum was significantly greater in the GASTR group compared to the CONT group (Table 2). The size of the Auerbach ganglia in the jejunum in the gastrectomy-treated group increased, as evidenced by the greater area and mean Feret diameter (Table 2). In liver, the number of total cells, hepatocytes, hepatocyte nuclei and mononuclear hepatocyte nuclei significantly increased in the GASTR group (Table 3). The content of immature collagen in the liver tissue was also higher in the GASTR group than in the CONT group (Table 3; Figure 3B).

### 3.2. Immunolocalization

The immune reactions with anti-cadherin antibodies in both examined parts of the digestive tract (the duodenum and the middle part of the jejunum), were similar in both groups. The reactions were continuous and the cadherins were observed on the basal layer of the epithelium, thus ensuring the maintenance of proper cell-cell contacts, however the intensity of cadherin expression was stronger after gastrectomy (Figure 4B,C).

**Figure 5:**
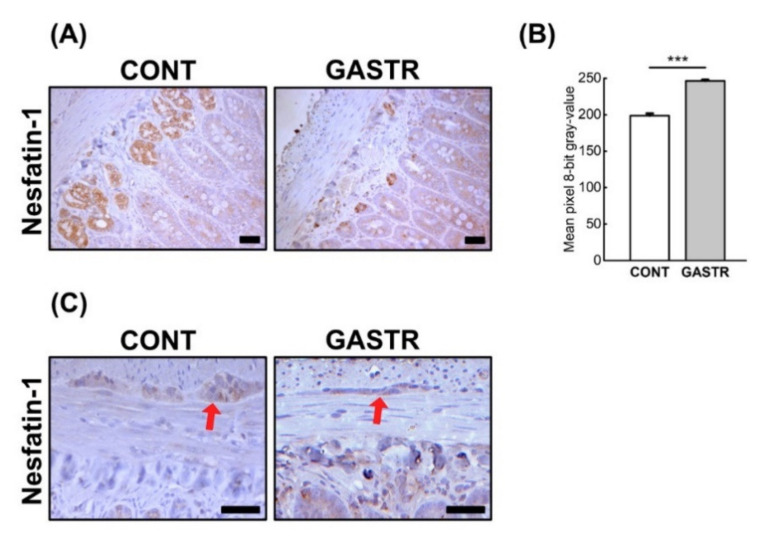
(**A**) Representative photomicrographs of the immunohistochemical reactions for nesfatin-1 in the jejunal crypts and (**C**) the Auerbach plexus (red arrow). Sections were developed in 3,3′-diaminobenzidine tetrahydrochloride (DAB); counterstaining was performed with Mayer’s hematoxylin. All the scale bars represent 50 μm. (**B**) The intensity of expression of nesfatin-1 in the jejunum, measured by the quantitative assessment of mean pixel intensity values in the photomicrographs converted to 8-bit grayscale images. Scale from 0 (white pixel) to 255 (black pixel); the lower the pixel value, the higher the intensity of immunohistochemical reaction. Graph shows mean ± standard error. Significance was established using a two-tailed Student’s *t*-test (normally distributed data), Welch’s test (normally distributed data with unequal variances) or the Mann–Whitney test (for pairwise comparisons with at least one non-normally distributed dataset); *** *p* < 0.001.

Immunolocalization of the nesfatin-1 peptide showed that nesfatin-1 IR cells were observed in both the duodenum and jejunum. In the duodenum of CONT rats, IR cells were mainly observed in Paneth cells within all crypts, with less nesfatin-1 immunostaining cells observed in the crypts. The duodenum of GASTR rats was also characterized by an immunostaining reaction in the Paneth cells of a few of the crypts. Moreover, the intensity of nesfatin-1 expression in the duodenum of gastrectomized rats was significantly weaker than that observed in the CONT rats. In the jejunum, the staining intensity was strong in the Paneth cells within the majority of the crypts and weak in the enterocytes in both groups (brown staining, Figure 3A); however, a weaker reaction was observed in the GASTR rats (Figure 5B). Immunolocalization of the nesfatin-1 peptide showed that nesfatin-1 IR cells were observed in both the Meissner and Auerbach plexuses in the duodenum and the jejunum. The intensity of the reaction in the IR cells of the Meissner plexus was comparable in both groups, while the expression of nesfatin-1 in the Auerbach plexuses was higher in the CONT rats compared to the GASTR rats (red arrow; Figure 5C).

**Figure 6:**
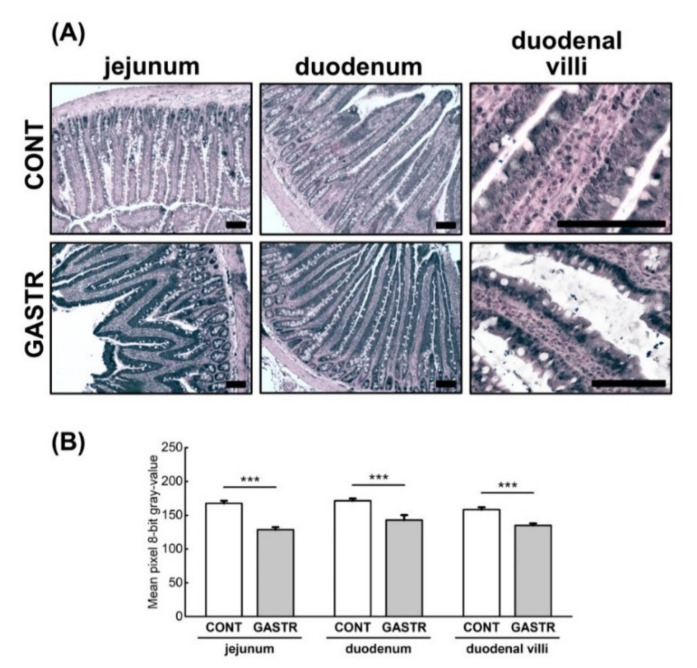
(**A**) Representative photomicrographs of the immunohistochemical reactions for zonula occludens 1 (ZO-1). Sections were developed in 3,3′-diaminobenzidine tetrahydrochloride with metal enhancer; counterstaining was performed with Nuclear Fast Red. All the scale bars represent 100 μm. (**B**) The intensity of expression of ZO-1, measured by comparison of the pixel brightness values in the microscopic images converted to 8-bit grayscale. The higher the pixel value, the lower the intensity of immunoreactions. Graph shows mean ± standard error. Significance was established using a two-tailed Student’s *t*-test (normally distributed data), Welch’s test (normally distributed data with unequal variances) or the Mann–Whitney test (for pairwise comparisons with at least one non-normally distributed dataset); *** *p* < 0.001.

The expression of ZO-1 (tight junction protein-1), which is one of the proteins involved in signal transduction at cell–cell junctions, was higher in the crypts and epithelial villi of the duodenum and the jejunum of the GASTR rats compared to the CONT rats (Figure 6A,B).

**Figure 7:**
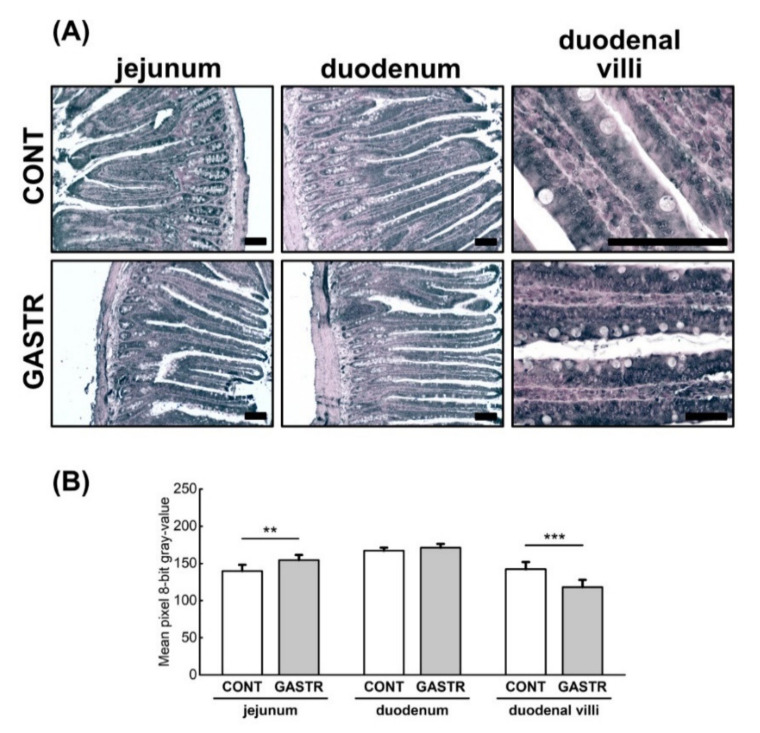
(**A**) Representative photomicrographs of the immunohistochemical reactions for occludin. Sections developed in 3,3′-diaminobenzidine tetrahydrochloride with metal enhancer; counterstaining performed with Nuclear Fast Red. All the scale bars represent 100 μm. (**B**) The intensity of expression of occludin measured by the comparison of the pixel brightness values in the microscopic images converted to 8-bit grayscale. The higher the pixel value, the lower the intensity of immunoreactions. Graph shows mean ± standard error. Significance was established using a two-tailed Student’s *t*-test (normally distributed data), Welch’s test (normally distributed data with unequal variances) or the Mann–Whitney test (for pairwise comparisons with at least one non-normally distributed dataset); ** *p* < 0.01; *** *p* < 0.001.

The expression of occludin, another integral plasma membrane protein located at the tight junctions, was of a similar intensity in the duodenum in both groups when analyzed across the whole epithelium. However, when occludin expression was analyzed separately for villi and crypts, a stronger intensity was noted in the villi from the GASTR rats compared to the CONT rats (Figure 7A,B). The opposite effect was observed in the jejunum, where the intensity of occludin expression was weaker in the GASTR rats compared to the CONT rats, irrespective of the part analyzed.

**Figure 8:**
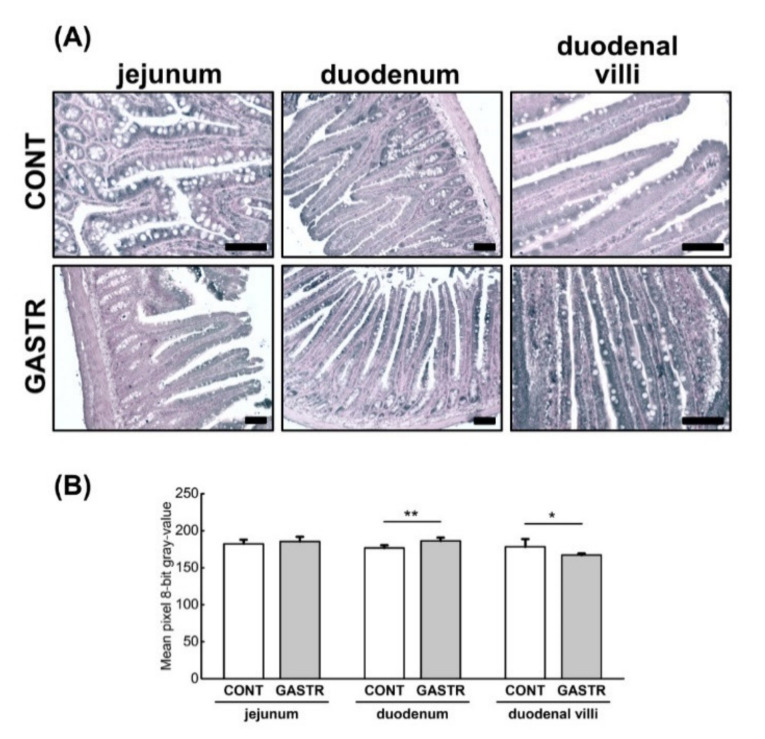
(**A**) Representative photomicrographs of the immunohistochemical reactions for marvelD3. Sections were developed in 3,3′-diaminobenzidine tetrahydrochloride with metal enhancer; counterstaining was performed with Nuclear Fast Red. All the scale bars represent 100 μm. (**B**) The intensity of expression of marvelD3, measured by the quantitative assessment of mean pixel intensity values in the photomicrographs converted to 8-bit grayscale images. Scale from 0 (white pixel) to 255 (black pixel); the lower the pixel value, the higher the intensity of immunohistochemical reaction. Graph shows mean ± standard error. Significance was established using a two-tailed Student’s *t*-test (normally distributed data), Welch’s test (normally distributed data with unequal variances) or the Mann–Whitney test (for pairwise comparisons with at least one non-normally distributed dataset); * *p* < 0.05; ** *p* < 0.01.

The expression of marvelD3, a tight junction protein that, like occludin, contains a conserved four-transmembrane MAL and related proteins for vesicle trafficking and membrane link (MARVEL) domain, was of a weaker intensity throughout the whole duodenal epithelium in the GASTR rats. However, when the expression was assessed only in the villi, the intensity of marvelD3 expression was stronger in villi from the GASTR rats compared to the CONT rats (Figure 8A,B). No differences in marvelD3 expression were observed in the jejunum (Figure 8).

**Figure 9:**
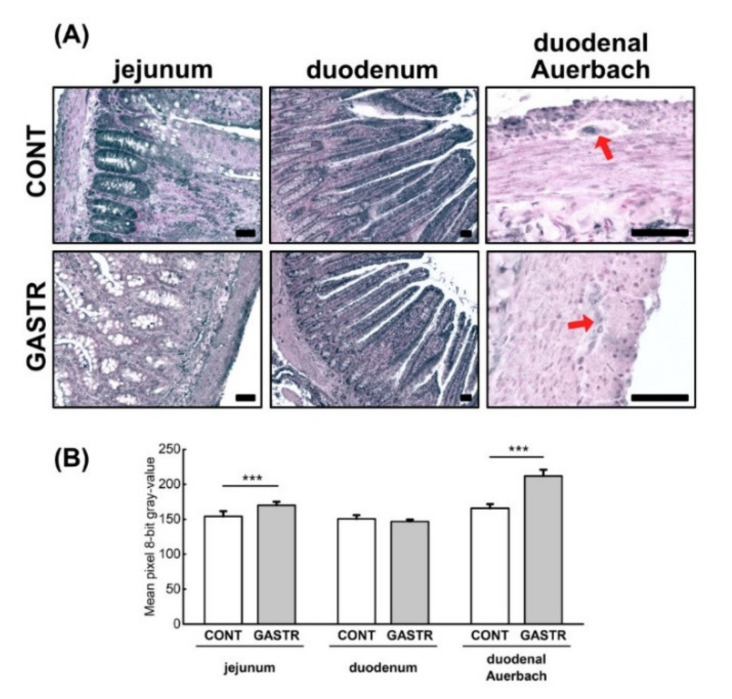
(**A**) Representative photomicrographs of the immunohistochemical reactions for ghrelin in the jejunum, duodenum and duodenal Auerbach plexus (red arrow). Sections developed in 3,3′-diaminobenzidine tetrahydrochloride with metal enhancer; counterstaining performed with Nuclear Fast Red. All the scale bars represent 100 μm. (**B**) The intensity of expression of ghrelin, measured by the comparison of the pixel brightness values in the microscopic images converted to 8-bit grayscale. Scale from 0 (white pixel) to 255 (black pixel); the lower the pixel value, the higher the intensity of immunohistochemical reaction. Graph shows mean ± standard error. Significance was established using a two-tailed Student’s *t*-test (normally distributed data), Welch’s test (normally distributed data with unequal variances) or the Mann–Whitney test (for pairwise comparisons with at least one non-normally distributed dataset); *** *p* < 0.001.

The expression of ghrelin, a hormone produced in the gastrointestinal tract, was similar in the duodenum of both groups (Figure 9A,B). Ghrelin expression was decreased in the jejunum of GASTR rats compared to that observed in CONT rats. Moreover, ghrelin expression was decreased in the enteric nervous system of the rats subjected to gastrectomy, irrespective of the part of the intestinal tract (duodenal Auerbach, Figure 9A,B).

**Figure 10:**
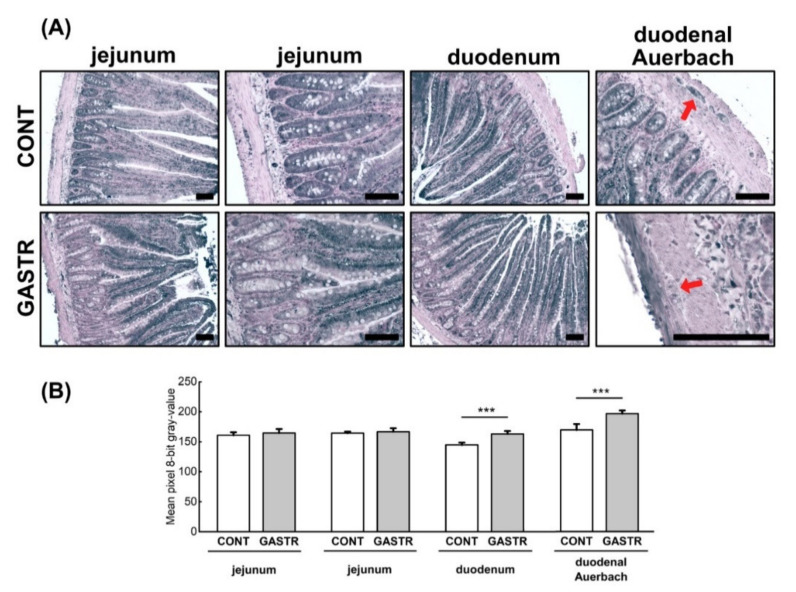
(**A**) Representative photomicrographs of the immunohistochemical reactions for leptin in the jejunum, duodenum and duodenal Auerbach plexus (red arrow). Sections were developed in 3,3′-diaminobenzidine tetrahydrochloride with metal enhancer; counterstaining was performed with Nuclear Fast Red. All the scale bars represent 100 μm. (**B**) The intensity of expression of ghrelin, measured by the comparison of the pixel brightness values in the microscopic images converted to 8-bit grayscale. Scale from 0 (white pixel) to 255 (black pixel); the lower the pixel value, the higher the intensity of immunohistochemical reaction. Graph shows mean ± standard error. Significance was established using a two-tailed Student’s *t*-test (normally distributed data), Welch’s test (normally distributed data with unequal variances) or the Mann–Whitney test (for pairwise comparisons with at least one non-normally distributed dataset); *** *p* < 0.001.

The expression of leptin, another hormone involved in the regulation of energy balance through the inhibition of hunger, was decreased in the duodenum after gastrectomy (Figure 10A,B). This decrease was observed in the crypts, epithelium villi and enteric nervous system (Figure 10A,B). In the jejunum, leptin expression was weaker in the crypts and stronger in the villi of rats from the GASTR group, whereas rats from the CONT group showed stronger leptin expression in the crypts versus in the villi. Thus, the overall total intensity of leptin expression was not different between groups (Figure 10A,B).

**Figure 11:**
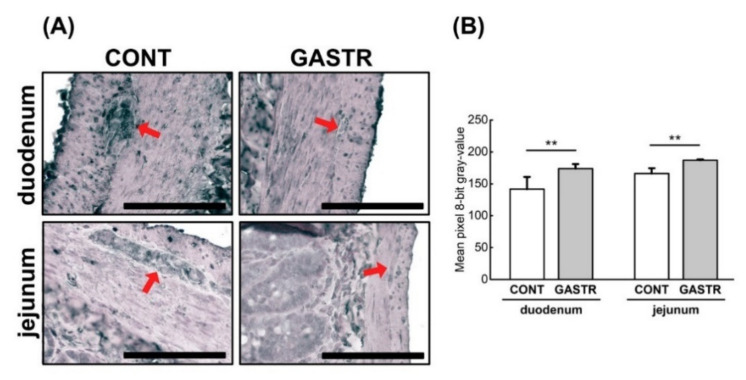
(**A**) Representative photomicrographs of the immunohistochemical reactions for vasoactive intestinal peptide (VIP) in Auerbach plexuses (red arrow) of the duodenum and the jejunum. Sections were developed in 3,3′-diaminobenzidine tetrahydrochloride with metal enhancer; counterstaining was performed with Nuclear Fast Red. All the scale bars represent 100 μm. (**B**) The intensity of expression of VIP, measured by the quantitative assessment of mean pixel intensity values in the photomicrographs converted to 8-bit grayscale images. Scale from 0 (white pixel) to 255 (black pixel); the lower the pixel value, the higher the intensity of immunohistochemical reaction. Graph shows mean ± standard error. Significance was established using a two-tailed Student’s *t*-test (normally distributed data), Welch’s test (normally distributed data with unequal variances) or the Mann–Whitney test (for pairwise comparisons with at least one non-normally distributed dataset); ** *p* < 0.01.

The expression of a vasoactive intestinal peptide (VIP) in the intestine, which induces smooth muscle relaxation, was decreased in the enteric nervous system after gastrectomy in both the duodenum and the jejunum (Figure 11A,B).

## 4. Discussion

Total or partial resection of the stomach involves changes in the functioning of the GIT. Patients after total gastrectomy can lose body weight due to dietary restrictions and disturbances in digestion and nutrient absorption. Dietary restrictions most often result from the lack of hunger, an early feeling of fullness and eating too little food [28]. In morbidly obese patients, despite the fact that bariatric surgery can lead to liver dysfunction and exocrine pancreatic insufficiency, it generally improves the quality of life and reduces mortality. Furthermore, as the intestine shows enteroplasticity, which is a manifestation of the ability to adapt to various conditions, bariatric surgery has consequences in its physiology and morphology as a result of the change in cell turnover [29]. The process of enterocyte proliferation can involve changes in villus height, crypt depth, mucosal surface area and intestinal mass, which all indicate morphological adaptation of the intestine [30]. In addition to morphological changes, enteroplasticity also includes adaptations within the nervous system as well as endocrine and nutrient signaling [14]. However, enteroplasticity can have both positive and negative consequences [31,32]. Seeley et al. hypothesized that enteroplasticity underlies the benefits of bariatric surgery [14]. Changes in neuronal innervation or neuronal activity can result in an increase in enteroendocrine cell number or sensitivity to stimuli and enhancement of nutrient absorption via an increase in villi number and/or length and/or depth of crypt as well as stimulation of intracellular signaling processes via an increase in nutrient transport or production of digestive products [14]. In the present study, gastrectomy caused a decrease in submucosa thickness and the number of active crypts in the duodenum. On the other hand, an increase in the thickness of the mucosa, submucosa and myenteron was observed in the jejunum, with a simultaneous reduction in enterocyte number. The changes in the jejunum were also accompanied by increased cell proliferation. Moreover, immature collagen fibers were observed in both the duodenum and the jejunum. In the current study, small changes in morphology were observed within the duodenum following gastrectomy, while in the jejunum, hypertrophy occurred. Previous studies observed no changes in intestinal morphology after gastrectomy in rats and mice [33,34]. However, morphological changes were observed following different types of surgeries involving GIT manipulations in rodent models. Dib et al. [10] observed a significant reduction in duodenal villi length and thickness of the mucosa and myenteron after biliopancreatic diversion and gastric sleeve procedures (Scopinaro method) in Wistar rats. In a rat model that underwent one-anastomosis (mini) gastric bypass (MGB) surgery, the alimentary limb was hyperplasic with a larger diameter, longer villi and deeper crypts [35]. This hyperplasia was only limited to the new food path and insufficient to compensate for the malabsorption [35]. When the stomach is left intact and the upper gut is bypassed (duodenojejunal bypass), atrophy in the bypassed limb and hyperplasia in the portion of the jejunum exposed to nutrients were observed [11]. Similarly, intestinal proliferation has been observed in previous studies after Roux-en-Y gastric bypass (RYGB), where significant increases in cell proliferation, intestine width, villus height and crypt depth were observed in the alimentary and common intestinal limbs [12,13]. Other surgeries involving intestinal manipulations, i.e., placement of a duodenal endoluminal sleeve and ileal interposition, also demonstrated intestinal hyperplasia in rodents [36,37]. The above-mentioned results indicate that functional elimination of one part of the GIT may cause a compensatory response in the remaining parts, involving some form of morphological adaptation. Thus, intestinal resection results in hypertrophy of the remaining intestine. This hypertrophy is a result of intestinal hyperplasia, which is associated with a higher rate of cell proliferation in the crypts and an increase in crypt depth and villi height [38,39]. These morphological changes probably depend on the length of the resected intestine and the type of surgery manipulation [39]. However, intestinal cell proliferation occurs after surgical manipulation in the intestine while vertical sleeve gastrectomy (VSG) does not affect intestinal morphology in experimental animals [33,34]. In light of these results, the hypertrophy combined with an increase in cell proliferation, immature collagen content and the simultaneous decrease in the number of enterocytes observed in the jejunum in the current study can be explained by the range of the performed surgery, and further studies are required to understand underlying mechanisms.

Increased villus height and improved nutritional intake may not be accompanied by an increase in individual cell width and height [40]. This suggests that intestinal adaptation comprises not only changes to the number of cells but also to the quality of the enterocytes [29]. Following adaptation, the intestinal enterocytes are more functional and more capable of digesting and absorbing nutrients. This may be confirmed by an increase in enzymatic activity within the intestine. An increase in the activity of intestinal brush border enzymes has previously been observed after surgical manipulations of the stomach [41]. An increase in the protein content and enzymatic activity of the duodenum and jejunum, in combination with slight morphological changes, may testify to the functional adaptation of the intestine after gastrectomy [41]. It can indicate that neither intestinal malabsorption nor mechanical restriction are the only mechanisms involved in reduced caloric intake or decreased body weight after bariatric surgery [42]. The lack of reduction in body weight in gastrectomized subjects could be caused by the lack of disturbances in the sodium-dependent absorption of glucose. Whether it was one of the mechanisms responsible for the lack of reduction in body weight following gastrectomy noted in our study should not be excluded. One should also remember that rats are able to adapt to specific metabolic situations by modulating meal frequency [42].

As previously mentioned, enteroplasticity can be associated with changes in the nervous system within the GIT [14]. The small intestine is supplied with fibers of the autonomic nervous system (ANS) and the enteric nervous system (ENS). Both systems are involved in the control of intestinal motility, blood flow, mucosal transport and secretions, as well as endocrine and immune functions. Gautron et al. confirmed the hypothesis that enteroplasticity is reliant on changes in the nervous innervation of the GIT [43]. They reported the loss of innervation by the vagus nerve within the stomach and intestine after surgical anastomoses and no changes in innervation of the intact intestinal segments and liver [43]. Morphological abnormalities within vagal nerve fibers were also observed, which were mainly associated with the myenteric plexus of the stomach [43]. Gastrectomy leads to disappearance of neural gastric reflexes, rapid gastric emptying, asynchrony between gastric emptying and bilio-pancreatic secretion and denervation of the pancreas due to dissection of lymph nodes and truncal vagotomy [44,45,46]. These changes contribute to the development of exocrine pancreatic insufficiency (EPI), which is one of the possible mechanisms of reduced digestion and malabsorption following gastric surgery. Proper integrity of the gastrointestinal–pancreatic complex is essential for an adequate process of digestion.

In the present study, myenteric (Auerbach) and submucosal (Meissner) plexus parameters were investigated. Gastrectomy was found to increase the number of Meissner ganglia in the duodenum as well as the size of the Auerbach ganglia in the jejunum. These changes in GIT innervation or neuronal activity could influence GI peptide secretion. In a study by Hansen et al. [47], glucagon-like peptide-1 (GLP-1) secretion was inhibited by sympathetic nervous system activation, whereas the extrinsic vagal supply had no effect. Instead, neurotransmitters from the intrinsic enteric nervous system significantly increased GLP-1 secretion and, thus, are thought to play a role in the GLP-1 secretion elicited by local reflexes [47]. It can therefore be assumed that changes in the ENS after gastrectomy affect enteroendocrine cell secretions.

The number and diversity of peptides and hormones derived from the GIT make it the largest endocrine organ of the body. These hormones can act directly on the GIT, including the ENS or other organs, after their transfer to the blood. The gastric endocrine cells release, among others, nesfatin-1 and ghrelin [48,49,50,51,52,53]. Nesfatin-1, which is also expressed in the duodenum, pancreas and colon [51,54,55], reduces food intake and is involved in the regulation of body weight [56] and inhibits gastric motility and emptying, duodenal motility and the vagally mediated stimulation of gastric acid secretion [56,57,58]. Ghrelin, an orexigenic peptide, increases food intake and decreases energy expenditure, altering body weight gain [59]. Intravenous administration of ghrelin dose-dependently increases gastric acid secretion and stimulates gastric motility [60]. Ghrelin-IR cells are found in the duodenum, jejunum, ileum, colon and pancreas in both rats and humans (except colon) [61,62]. The stomach is also a source of leptin [63], initially described as a hormone synthesized by adipose tissue [64]. Leptin mediates the regulation of energy balance, metabolism, neuroendocrine and immune function and development [65,66]. In the GIT, leptin is involved in intestinal transport and enterocyte metabolism [67,68]. Since gastric-derived hormones are involved in various activities throughout the GIT, the elimination of their impact/function following total gastrectomy may be important not only for GIT functioning but also for the control of food intake, glycemia and other actions.

The epithelium of the small intestine contains less than 1% endocrine cells, which are the source of many peptides including secretin, cholecystokinin (CCK), glucose-dependent insulinotropic polypeptide (GIP), GLP-1, glucagon-like peptide-2 (GLP-2), pro-satiety hormone peptide YY (PYY), somatostatin, secretin, neurotensin, motilin and ghrelin [69]. According to research, the expression and secretion of hormones in the small intestine depend on systemic factors related to metabolic status as well as locally acting factors. Changes in the number or density of endocrine cells were observed after various anastomosis surgeries. In a study by Mumphrey et al., an increase in the number, but not density, of CCK-, GLP-1-, serotonin- and neurotensin-expressing enteroendocrine cells in the rat intestine was observed [70]. RYGB surgery has been shown to have no effect on the density of ghrelin-, CCK-, neurotensin-, secretin- and serotonin-producing cells in other studies [71]. The increase in the number of endocrine cells, with no modifications to cell density, may be a consequence of the hyperplasia of the alimentary limb. In turn, an increased density of GLP-1-, PYY- and GIP-positive cells after RYGB was observed [71]. Changes in enteroendocrine cell number or density after one-anastomosis gastric bypass (OAGB) surgery have previously been described [35]. Hyperplasia caused an increase in the number of GLP-1- and GIP-secreting cells, thus contributing to the increase in their secretion. After SG, an increase [33], or a decrease [34], in the density of cells expressing GLP-1 was observed.

In the present study, we did not assess the number and density of endocrine cells but rather the immunohistochemical expression of nesfatin-1, ghrelin, leptin and VIP in the duodenum and jejunum. IR cells for nesfatin-1, ghrelin and leptin were observed in the villi, crypts and ENS, in both the duodenum and jejunum, with IR cells for VIP observed in the ENS. These results are partly consistent with the results of previous studies in relation to the location of these hormones in the intestine [55,61,62,72,73,74]. We observed a decrease in the expression of nesfatin-1 and leptin in the duodenum mucosa and a decrease in ghrelin expression in the jejunum mucosa. These changes may be important for intestine function since the above-mentioned peptides are involved in GIT activities. Leptin may play a role in growth (proliferation of mucosal epithelial cells), nutrient absorption by enzymatic activity in the brush border of the enterocytes and gut motility [74]. Nesfatin-1 plays a role in enzyme activation, nutrient absorption and protection of the intestinal walls [55], and ghrelin is involved in the regulation of gastrointestinal motility [75]. Perhaps this is partly due to their effect on the release of other hormones. Nesfatin-1 has been found to stimulate GLP-1, GIP and CCK and suppress PYY expression and secretion in vitro [76,77]. Unfortunately, literature concerning the consequences of gastrectomy on the expression of the hormones assessed in the present study is very limited. Teive et al. [78] observed no significant changes in the number of ghrelin-positive cells in the duodenum of rats after SG.

We found that gastrectomy decreased the immunohistochemical reaction for leptin and VIP in the submucosal and myenteric plexuses in the duodenum, for nesfatin-1 in the myenteric plexuses in the duodenum and jejunum and for VIP in the ENS in both the duodenum and jejunum. Submucosal neurons of the ENS are the regulators of mucosal function, while myenteric neurons participate in the regulation of GIT motility. VIP, localized in the myenteric and submucosal neurons and nerve terminals in the GIT [72], stimulates anion secretion from the enterocytes [79], contracts [80], and relaxes [81] GIT smooth muscles and modulates epithelial paracellular permeability via regulation of the expression and function of tight junction proteins [82]. VIPergic pathways were found to increase the expression of ZO-1 in colonic epithelium in an in vitro study, which is, in turn, associated with reduced epithelial paracellular permeability [82]. However, in the present study, a stronger immunohistochemical reaction for ZO-1 occurred in conjunction with a weaker reaction for VIP in the ENS. The presence of immunopositive neurons for nesfatin-1, ghrelin and leptin in the ENS indicated that these hormones are also implicated in the modulation of intestinal motility and other functions. Thus, changes in their expression in the ENS after gastrectomy may affect the function of the GIT. Nesfatin-1 immunopositive neurons and nervous fibers in the internal submucosal plexus of the duodenum, external submucosal plexus of the ileum and myenteric plexuses in the colon were observed by Varricchio et al. in Casertana pigs [73], while Gonkowski et al. did not observe any nesfatin-1 immunopositive neurons or nervous fibers in the enteric neurons in dog duodenum [54]. Leptin has been shown to have an impact on enteric nitrergic neurons and intrinsic primary afferent neurons as well as on the activation of myenteric and submucosal neurons [83,84]. In turn, ghrelin stimulates motility of the small intestine through intrinsic cholinergic neurons [75]. However, the role of nesfatin-1 in the ENS is not well understood [85]. As observed in the present study, neurochemical changes in neurons are the main manifestation of the plasticity of the ENS.

The intestinal epithelium plays an important role in separating the luminal contents from surrounding tissues [86]. The properties of this barrier are achieved by the formation of a complex multi-protein network between the epithelial cells including tight junctions, adhering junctions and gap junctions [87]. These proteins belong to the group of proteins responsible for the properties of the epithelial barrier in the gut. Gap junctions link the cytoplasm of adjoining cells and provide a pathway for intercellular exchange of ATP, ions and fluids [87]. They are required for maintaining cellular function and homeostasis. The mechanical integrity of the epithelium barrier is maintained by gap and adhering junctions, which ensure adhesive contact between cells [88]. Tight junctions act as a barrier against the extracellular environment, regulate paracellular permeability and polarity and modulate intracellular and intercellular signaling and transport [88,89,90]. Tight junction proteins are classified into three types of proteins, including transmembrane, cytoskeletal and cytoplasmic plaque proteins [91]. Occludin and marvelD3, evaluated in the present study, are classified as transmembrane proteins, whereas ZO-1 is a cytoplasmic plaque protein. Transmembrane proteins penetrate the cellular membrane and influence the passage of certain substances, paracellular transport and permeability [89]. Occludin is a primary transmembrane protein which interacts with cytoplasmic plaque proteins such as ZO-1, maintaining cell surface polarity [92]. In turn, ZO-1 interacts with other transmembrane proteins, claudin and junction adhesion molecule A (Jam A) [93]. ZO-1 protein modulates the structure of tight junctions, paracellular permeability and gene expression [93].

Assessment of the intestinal barrier after surgical manipulation within the GIT has only been reported in very few previous studies. In mice, SG modified intestinal permeability in both the small and large intestines in a differential manner. SG decreased paracellular and transcellular permeability measured ex vivo in biopsies of the jejunum. These jejunal changes were associated with higher mRNA expression of Jam A and occludin, whereas ZO-1 mRNA expression was unchanged [91]. In turn, ex vivo paracellular and transcellular permeabilities were increased in the distal colon after SG, whereas expression of occludin, Jam A and ZO-1 mRNAs was not significantly altered. However, SG increased paracellular permeability in vivo, despite the reduced jejunal permeability observed ex vivo [94]. Unfortunately, these results are difficult to interpret. Casselbrant et al. [95] observed increased expression of several tight junction proteins in the proximal small intestinal mucosa of humans after RYGB surgery, suggesting decreased paracellular permeability. The increased expression of occludin in the mucosa of the alimentary and common limbs after duodenojejunal bypass, observed by Yang et al. [96], is consistent with decreased permeability. These changes were also accompanied by an increase in villus height and crypt depth in both limbs, indicating epithelial proliferation. In the present study, we investigated the immunohistochemical expression of occludin, ZO-1 and marvelD3 proteins in the duodenum and jejunum. We observed that gastrectomy resulted in increased expression of both occludin and marvelD3 in the duodenal villi and increased ZO-1 expression in the villi and crypts of both the duodenum and the jejunum. These results indicate strengthening of the epithelial barrier function, but they are not associated with significant changes in intestinal morphology. However, an increase in the proliferative index in the jejunum may be an adaptive mechanism to maintain the integrity of the epithelial barrier. Thus, it is very likely that the adapted intestine not only has more enterocytes but also improved enterocytes, which are more capable of performing their function.

The effect of gastric surgery on the liver is still controversial. On the one hand, gastrectomy reduces liver lipid accumulation, glycogen content and gluconeogenic gene expression [97], and improves steatosis, steatohepatitis and fibrosis [98]; on the other hand, it can reveal liver dysfunction [99]. In the present study, changes in liver structure were also observed. These abnormalities are consistent with the literature; however, any correlation between these changes, general condition, selected biochemical parameters and the extent of the resection requires further controlled clinical and experimental studies [100,101]. 

Studies on rat models indicate an ambiguous effect of gastrectomy on body weight. The presented results concerning the lack of significant changes in body weight are consistent with reports of other authors who found no significant effect of gastrectomy on the body weight of rats [26,46,47]. Nevertheless, the tendency to gain weight more slowly has occurred [47]. On the other hand, there are studies that prove weight loss in rats after gastrectomy [102,103]. Loss of weight after gastrectomy leads to reduction in stomach volume, which may reduce food intake in rats. However, after an initial reduction in food intake after gastrectomy, total daily food intake can be restored by increasing meal numbers at a decreased meal size [104]. This indicates an adaptation of meal pattern to the reduced stomach volume. One should also remember that rats are able to adapt to specific metabolic situations by modulating meal frequency [105]. The lack of reduction in body weight in gastrectomized subjects could be caused by a lack of disturbances in the sodium-dependent absorption of glucose. Whether it was one of the mechanisms responsible for the lack of reduction in body weight following gastrectomy noted in our study should not be excluded. Gastrectomy leads, inter alia, to a reduction in ghrelin levels [102,103,106,107], and an elevation [107,108,109], or reduction in nesfatin-1 level [110]. Nesfatin-1 as an anorexigenic peptide and ghrelin as an orexigenic peptide affect food intake. Reduction in ghrelin level as well as a rise in nesfatin-1 level may be responsible for reduction in body weight after gastrectomy [102,108]. Regarding nesfatin-1, an increase in its preoperative levels suggests that sources other than the stomach provide a substantial amount of this hormone. However, the research indicates that ghrelin is an unlikely candidate to explain the effect of gastrectomy on body weight in rodents. This is evidenced by the fact that ghrelin-deficient mice are just as susceptible to gastrectomy-induced weight loss as wild-type controls [111]. Our research indicated a reduction in serum ghrelin level by 76% and an increase in serum nesfatin-1 level by 40% [107], but these changes were not associated with weight loss. The lack of influence of gastrectomy on body weight, as observed by us and by other authors, may indicate that hormonal changes resulting from gastrectomy are not the only ones responsible for body weight changes in the post-operative period in rats. It is highly probable that the lack of differences in body weight between the CONT and GASTR rats is due to the increased activity of digestive enzymes of the pancreas and intestine, which we previously reported [41].

Bariatric animal models, including rodents, are still significantly helpful in the collection of both qualitative and quantitative data concerning post-operative changes after gastrectomy [42]. While is not possible to directly extrapolate the numerous obtained data to human physiology, rat studies help to understand, step by step, all changes in the physiology and morphology of the GIT. Therefore, although the presented study has some limitations such as the lack of a detailed report of body weight gain or serum hormonal analysis or basal serum biochemical parameters, in our opinion, the obtained results can be clinically helpful and show that animal studies are a needed research tool, and especially that there are many similarities between rat model and humans, including changes in the post-operative profile of gut hormones, bile acids and metabolic effects of underwent bariatric surgery [112,113].

## 5. Conclusions

In conclusion, gastrectomy leads to changes in the small intestine morphology and the expression of some hormones and tight junction proteins. These changes testify to intestinal enteroplasticity, associated with changes in the GIT condition. The results obtained suggest that more complicated regulatory mechanisms are involved, over and above compensatory mucosal hypertrophy alone. The higher emptying rate of the stomach and diminished secretory capacity as well as a change in nutrients may modify the action of enteric neurons and enteroendocrine cells in the small intestine. Moreover, functional changes in the mucosa and ENS could be responsible for the altered intestinal barrier and hormonal responses following gastrectomy.

## Figures and Tables

**Table 1:** The histomorphometrical parameters of the duodenum and jejunum in male Wistar rats in the CONT (sham-operated) and GASTR (subjected to gastrectomy) groups.

Parameter	Duodenum		Jejunum	
	CONT	GAST	CONT	GAST
Myenteron longitudinal lamina thickness, μm	34.4 ± 8.85	29.2 ± 6.06	15.7 ± 4.6	27.2 ± 3.0 ***
Myenteron transversal lamina thickness, μm	52.5 ± 12.1	46.0 ± 9.1	22.7 ± 3.7	40.7 ± 3.7 ***
Submucosa thickness, μm	40.3 ± 8.2	25.4 ± 7.8 **	15.4 ± 7.6	31.7 ± 5.3 **
Mucosa thickness, μm	885 ± 73	825 ± 68	723 ± 31	825 ± 68 **
Villus length, µm	628 ± 34	622 ± 53	492 ± 59	460 ± 43
Villus thickness, µm	81.4 ± 10.3	81.8 ± 7.8	76.0 ± 9.8	81.3 ± 13.5
Total number of villi, /mm	9.1 ± 1.5	8.4 ± 0.8	9.9 ± 0.9	9.1 ± 1.0
Villus epithelium thickness, µm	30.9 ± 4.7	31.7 ± 4.4	28.9 ± 5.5	31.0 ± 6.7
Enterocyte number, /100 µm of villus	13.5 ± 1.4	14.8 ± 1.4	16.2 ± 1.9	13.5 ± 1.8*
Total crypts number, /mm	13.2 ± 2.7	11.6 ± 1.6	16.9 ± 3.9	17.3 ± 3.3
Active crypts number, /mm	4.4 ± 1.5	2.6 ± 0.7 *	5.7 ± 1.2	5.4 ± 1.9
Inactive crypts number, /mm	8.8 ± 2.6	9.0 ± 1.7	10.3 ± 3.8	11.9 ± 4.0
Crypt depth, μm	168 ± 27	146 ± 30	148 ± 19	136 ± 17
Crypt width, µm	52.7 ± 9.1	55.3 ± 8.6	44.3 ± 10.4	47.1 ± 9.0
Intestine absorptive surface, µm^2^	4.2 ± 0.6	4.9 ± 1.0	3.9 ± 0.6	4.0 ± 0.5
Immature collagen, %	4.1 ± 2.2	7.6 ± 2.4 *	3.5 ± 1.5	10.8 ± 2.6 ***
Ki index	0.61 ± 0.06	0.68 ± 0.06	0.39 ± 0.13	0.56 ± 0.07 *
Ki number, /0.01 mm^2^ of the gland surface	9.08 ± 2.01	8.11 ± 1.08	6.6 ± 1.8	10.1 ± 2.8 *

Table shows mean ± standard deviation. Statistical significance: * *p* < 0.05; ** *p* < 0.01; *** *p* < 0.001. Significance was established using a two-tailed Student’s *t*-test (normally distributed data), Welch’s test (normally distributed data with unequal variances) or the Mann–Whitney test (for pairwise comparisons with at least one non-normally distributed dataset). Ki index—the number of proliferating cells in relation to all gland cells; Ki number—the number of proliferating cells.

**Table 2:** The histomorphometrical parameters of the enteric nervous plexuses in the duodenum and jejunum of the male Wistar rats in the CONT (sham-operated) and GASTR (subjected to gastrectomy) groups.

Parameter	Duodenum		Jejunum	
	CONT	GAST	CONT	GAST
Auerbach plexus				
Area, µm^2^	828 ± 143	656 ± 155	585 ± 125	979 ± 149 ***
Perimeter, µm	137 ± 67	131 ± 37	120 ± 55	179 ± 74
Mean Feret diameter, µm	41.0 ± 33.4	39.3 ± 40.9	36.2 ± 9.5	53.6 ± 12.1 *
Mean diameter, µm	25.4 ± 10.2	20.2 ± 9.3	12.9 ± 6.5	13.0 ± 5.1
Min diameter, µm	15.9 ± 4.6	11.8 ± 3.6	21.9 ± 10.1	24.7 ± 12.3
Sphericity	0.31 ± 0.19	0.30 ± 0.22	0.21 ± 0.18	0.13 ± 0.11
The number of the ganglia, /mm	5.5 ± 0.9	5.6 ± 1.2	3.0 ± 1.1	3.1 ± 0.6
Meissner plexus				
Area, µm^2^	384 ± 114	372 ± 142	355 ± 103	372 ± 158
Perimeter, µm	82 ± 25	82 ± 32	80 ± 22	79 ± 34
Mean Feret diameter, µm	24.6 ± 7.4	24.5 ± 9.5	24 ± 6.7	23.9 ± 10.2
Mean diameter, µm	20.8 ± 6.3	20.1 ± 6.3	19.3 ± 5.6	19.4 ± 7.2
Min diameter, µm	14.6 ± 4.9	14.2 ± 4.4	12.8 ± 4.5	13.7 ± 5.3
Sphericity	0.29 ± 0.19	0.31 ± 0.05	0.31 ± 0.18	0.33 ± 0.24
The number of the ganglia, /mm	1.5 ± 0.2	4.4 ± 0.8 ***	3.8 ± 0.9	3.9 ± 0.1

Table shows mean ± standard deviation. Statistical significance: * *p* < 0.05; *** *p* < 0.001. Significance was established using a two-tailed Student’s *t*-test (normally distributed data), Welch’s test (normally distributed data with unequal variances) or the Mann–Whitney test (for pairwise comparisons with at least one non-normally distributed dataset).

**Table 3:** The histomorphometrical parameters of the liver in male Wistar rats in the CONT (sham-operated) and GASTR (subjected to gastrectomy) groups.

Parameter	CONT	GAST
Total cell number, /mm^2^	2084 ± 189	2647 ± 138 ***
Total hepatocyte number, /mm^2^	1565 ± 75	2149 ± 55 ***
Total hepatocyte nucleus number, /mm^2^	1651 ± 65	2257 ± 62 ***
Mononuclear hepatocytes nucleus number, /mm^2^	1479 ± 119	2041 ± 157 ***
Binuclear hepatocytes nucleus number, /mm^2^	86 ± 28	108 ± 29
Non-hepatocyte cell number, /mm^2^	519 ± 99	488 ± 51
Immature collagen, %	15.8 ± 4.5	39.8 ± 5.3 ***

Table shows mean ± standard deviation. Statistical significance: *** *p* < 0.001. Significance was established using a two-tailed Student’s *t*-test (normally distributed data), Welch’s test (normally distributed data with unequal variances) or Mann–Whitney test (for pairwise comparisons with at least one non-normally distributed dataset).

## Data Availability

The data presented in this study are available on request from the corresponding author.

## Supplementary Materials

The following are available online at https://www.mdpi.com/2077-0383/10/2/272/s1, Figure S1: Representative pictures of the antibody control for all immunohistochemical reactions; Table S1: Descriptive statistics of the data and results of statistical analyses
