# Supplementary material for: Alterations in Small Intestine and Liver Morphology, Immunolocalization of Leptin, Ghrelin and Nesfatin-1 as Well as Immunoexpression of Tight Junction Proteins in Intestinal Mucosa after Gastrectomy in Rat Model
Source: J Clin Med. 2021 Jan 13;10(2):272. doi: 10.3390/jcm10020272 (PMC7828391; doi:10.3390/jcm10020272)
Supplement: Supplementary file 1 [file jcm-10-00272-s001.pdf]

Supplementary material

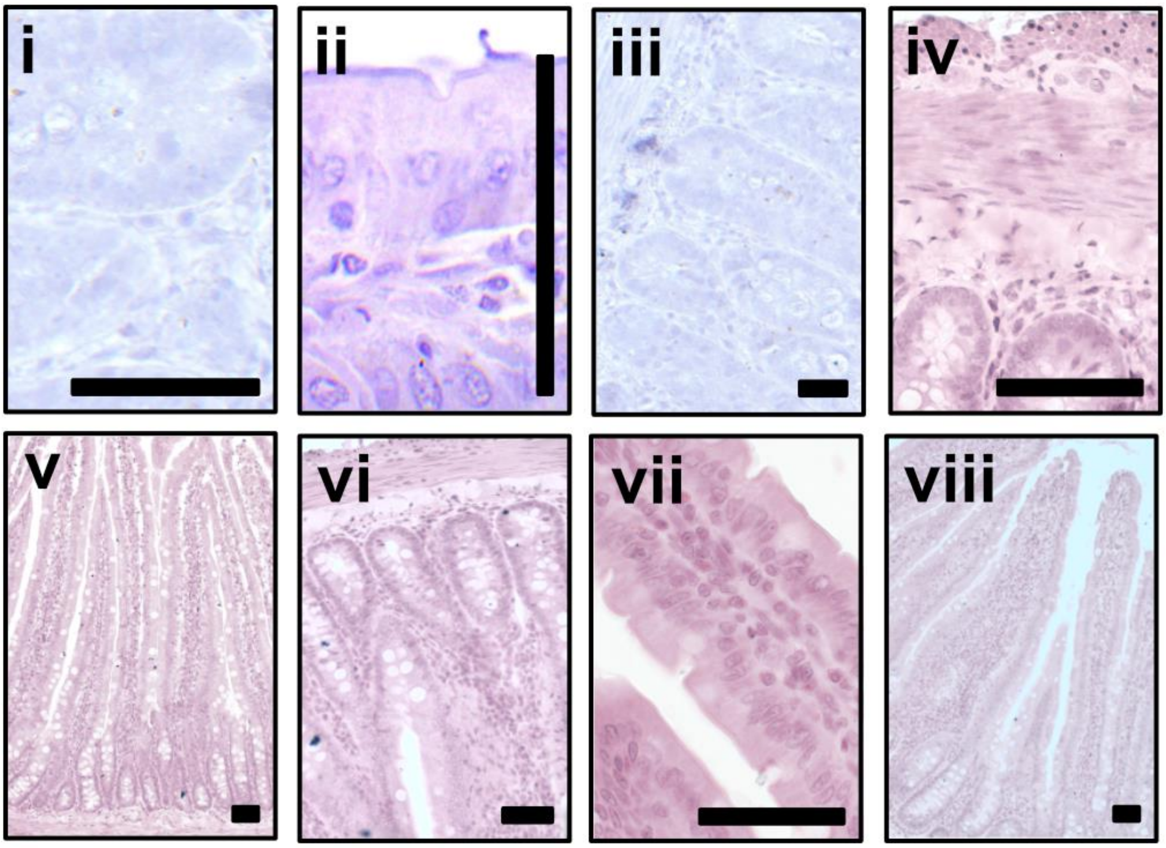

Figure S1. Representative pictures of the antibody control for all immunohistochemical reactions: (i) crypts in jejunum; (ii) enterocytes in jejunum; (iii) crypts in duodenum; (iv) plexus of enteric nervous system in duodenum; (v) villi in duodenum; (vi) crypts in duodenum; (vii) duodenal villus; (viii) duodenal villi. (i, ii, iii) Sections developed in 3,3'-diaminobenzidine tetrahydrochloride (DAB); counterstaining performed with Mayer's hematoxylin. (iv, v, v, vii, viii) Sections developed in 3,3'-diaminobenzidine tetrahydrochloride (DAB) with metal enhancer; counterstaining performed with Nuclear Fast Red (NFR). All the scale bars represent 50 μm.

Statistical analysis

Sample size (n = 6 per group) was calculated for a two-sided t-test of two groups with an α of 0.05 and power at 0.8, with standardized effect size of 1.8 [S1]. Available literature data on gastrectomy-subjected rats show that for these assumptions n = 6 sample size has a power of 80% to detect a change of 5-17% in villus height [S2-S5], a change of 6% in villus number [S2], and a change of 10-11% in crypt depth [S2, S4], assuming a 5% significance level [S6].

The results included in Table S1 are expressed as mean ± standard error and median (with Q1 and Q3), where Q1 and Q3 are the 25th and 75th percentile, respectively. Before the comparison of the means, the normality and homogeneity of variance was tested for all data with Shapiro-Wilk's test and Levene's test, respectively. The group comparison was performed by unpaired two-tailed Student's t-test (normally distributed data with equal variances), unpaired t-test with Welch's correction (normally distributed data with unequal variances) or the Mann Whitney test (for pairwise comparisons with at least one non-normally distributed dataset). Effect size for Student's and Welch's t-test was calculated using Cohen's *d*; for Mann–Whitney's test Cohen's *r* was determined [S7].

Supplementary References

S1. Festing, M.F.W. On determining sample size in experiments involving laboratory animals. *Lab. Anim.* **2018**, *52*, 341–350.
S2. Onishi, S.; Kaji, T.; Yamada, W.; Nakamee, K.; Machigashire, S.; …, Ieiri, S. Ghrelin stimulates intestinal adaptation following massive small bowel resection in parenterally fed rats. *Peptide* **2018**, *106*, 59–67.
S3. Stearns, A.T.; Balakrishnan, A.; Tavakkolizadeh, A. Impact of Roux-en-Y gastric bypass surgery on rat intestinal glucose transport. *Am. J. Physiol. Gastrointes. Liver Physiol.* **2009**, *297*, G950–957.
S4. Deveney, C.W.; Owen, R.L.; Deveney, K.; Reber, H.A.; Way, L.W. Effect of acid secretory capacity and chronic endogenous hypergastrinemia on pancreatic secretion and intestinal morphology in the rat. *Digest. Dis. Sci.* **1983**, *28*, 65–73.
S5. Li, L.; Wang, X.; Bai, L.; Yu, H.; Huang, X.; Huang, A.; Luo, Y.; Wang, J. The effects of sleeve gastrectomy on glucose metabolism and glucagon-like peptide 1 in Goto-Kakizaki rats. *J. Diabet. Res.* **2018**, *2018*, 1082561.
S6. Kilkenny, C.; Browne, W.J.; Cuthill, I.C.; Emerson, M.; Altman, D.G. Improving bioscience research reporting: The ARRIVE guidelines for reporting animal research. *PLoS Biol.* **2010**, *8*, e1000412.
S7. Fritz, C.O.; Morris, P.E.; Richler, J.J. Effect size estimates: Current use, calculations, and interpretation. *J. Exp. Psychol. Gen.* **2012**, *141*, 2–18.

Table S1. Descriptive statistics of the data and results of statistical analyses

Item	CONT		GASTR		Applied statistical test	*p*-value	Effect size
	Mean ± SE	Median (Q1; Q3)	Mean ± SE	Median (Q1; Q3)			
**Table 1.** The histomorphometrical parameters of the duodenum and jejunum							
Duodenum							
Myenteron longitudinal lamina thickness, µm	34.4 ± 3.61	36.9 (30.1; 41.0)	29.2 ± 2.47	29.5 (24.8; 34.0)	Student's t-test	0.262	
Myenteron transversal lamina thickness, µm	52.2 ± 4.92	52.6 (50.2; 61.1)	46.0 ± 3.71	45.3 (40.3; 46.7)	Student's t-test	0.318	
Submucosa thickness, µm	40.3 ± 3.34	41.8 (40.3; 42.6)	25.4 ± 3.18	27.4 (21.7; 28.8)	Student's t-test	0.009	1.70
Mucosa thickness, µm	885 ± 29.8	872 (834; 886)	825 ± 27.8	846 (755; 877)	Student's t-test	0.171	
Villus length, µm	628 ± 13.9	623 (603; 642)	622 ± 21.6	618 (598; 654)	Student's t-test	0.820	
Villus thickness, µm	81.4 ± 4.20	85.5 (74.1; 86.6)	81.8 ± 3.18	82.6 (74.9; 87.6)	Student's t-test	0.941	
Total number of villi, /mm	9.1± 0.61	9.1 (8.0; 9.4)	8.4 ± 0.33	8.4 (8.0; 9.1)	Student's t-test	0.337	
Villus epithelium thickness, µm	30.9 ± 1.92	30.9 (28.0; 33.2)	31.7 ± 1.80	31.7 (28.8; 34.4)	Student's t-test	0.767	
Enterocyte number/100 µm of villus	13.5 ± 0.57	13.7 (13.0; 14.2)	14.8 ± 0.59	14.9 (13.9; 15.4)	Student's t-test	0.139	
Total crypts number, /mm	13.2 ± 1.10	12.6 (11.9; 15.3)	11.6 ± 0.63	11.3 (10.9; 11/6)	Mann-Whitney's test	0.173	
Active crypts number, /mm	4.4 ± 0.61	4.4 (3.9; 4.8)	2.6 ± 0.29	2.8 (2.2; 3.0)	Student's t-test	0.024	1.40
Inactive crypts number, /mm	8.8 ± 1.06	9.5 (8.3; 10.7)	9.0 ± 0.69	8.8 (7.9; 10.1)	Student's t-test	0.878	
Crypt depth, µm	168 ± 11.0	163 (143; 196)	146 ± 12.2	153 (148; 163)	Mann-Whitney's test	0.575	

Crypt width, µm	52.7 ± 3.71	53<br>(46; 61)	55.3 ± 3.51	56<br>(49; 62)	Student's<br>t-test	0.622	
Intestine absorptive surface, µm^2	4.2 ± 0.24	3.9<br>(3.8; 4.9)	4.9 ± 0.41	4.7<br>(4.5; 5.5)	Mann-<br>Whitney's test	0.378	
Immature collagen, %	4.1 ± 0.90	3.5<br>(2.1; 6.4)	7.6 ± 0.98	7.6<br>(7.1; 9.5)	Student's<br>t-test	0.025	1.39
Ki index	0.61 ± 0.024	0.62<br>(0.61; 0.65)	0.68 ± 0.020	0.67<br>(0.62; 0.72)	Mann-<br>Whitney's test	0.093	
Ki number/0.01 mm^2 of the gland surface	9.08 ± 0.821	9.17<br>(7.13; 10.72)	8.11 ± 0.441	8.40<br>(7.48; 8.88)	Student's<br>t-test	0.332	
Jejunum							
Myenteron longitudinal lamina thickness, µm	15.7 ± 1.88	15.6<br>(11.9; 19.1)	27.2 ± 1.22	27.6<br>(25.0; 29.5)	Student's<br>t-test	<0.001	2.70
Myenteron transversal lamina thickness, µm	22.7 ± 1.51	23.8<br>(18.3; 25.2)	40.7 ± 1.55	41.5<br>(38.3; 42.7)	Student's<br>t-test	<0.001	4.44
Submucosa thickness, µm	15.4 ± 3.10	16.4<br>(7.8; 20.6)	31.7 ± 2.16	30.7<br>(30.1; 36.5)	Mann-<br>Whitney's test	0.008	0.76
Mucosa thickness, µm	723 ± 12.7	730<br>(696; 751)	825 ± 27.8	797<br>(782; 846)	Mann-<br>Whitney's test	0.005	0.81
Villus length, µm	492 ± 24.1	468<br>(447; 536)	460 ± 17.6	451<br>(435; 502)	Student's<br>t-test	0.308	
Villus thickness, µm	76.0 ± 4.00	77<br>(66; 85)	81.3 ± 5.51	75<br>(73; 95)	Mann-<br>Whitney's test	0.810	
Total number of villi, /mm	9.9 ± 0.37	10.0<br>(9.0; 10.8)	9.1 ± 0.41	9.5<br>(8.9; 9.7)	Mann-<br>Whitney's test	0.298	
Villus epithelium thickness, µm	28.9 ± 2.25	27.3<br>(25.1; 31.9)	31.0 ± 2.74	29.6<br>(28.5; 33.9)	Student's<br>t-test	0.566	
Enterocyte number/100 µm of villus	16.2 ± 0.78	16.8<br>(14.2; 17.4)	13.5 ± 0.73	13.2<br>(12.7; 13.8)	Student's<br>t-test	0.030	1.33
Total crypts number, /mm	16.9 ± 1.59	17.5<br>(14.9; 20.1)	17.3 ± 1.35	16.5<br>(14.8; 18.4)	Student's<br>t-test	0.852	
Active crypts number, /mm	5.7 ± 0.73	5.9<br>(5.1; 7.3)	5.4 ± 0.78	5.7<br>(4.6; 6.9)	Student's<br>t-test	0.785	

Inactive crypts number, /mm	10.3 ± 1.55	10.9<br>(9.6; 12.3)	11.9 ± 1.63	12.7<br>(7.7; 13.6)	Student's<br>t-test	0.494	
Crypt depth, µm	148 ± 7.8	153<br>(127; 162)	136 ± 6.9	138<br>(126; 151)	Student's<br>t-test	0.276	
Crypt width, µm	44.3 ± 4.24	43.3<br>(36.6; 49.3)	47.1 ± 3.67	46.6<br>(40.4; 51.4)	Student's<br>t-test	0.629	
Intestine absorptive surface, µm^2	3.9 ± 0.24	3.9<br>(3.6; 4.4)	4.0 ± 2.04	4.0<br>(3.6; 4.3)	Student's<br>t-test	0.760	
Immature collagen, %	3.5 ± 0.61	3.3<br>(2.1; 5.0)	10.8 ± 1.08	10.3<br>(9.9; 12.8)	Welch's t-test	<0.001	3.14
Ki index	0.39 ± 0.053	0.39<br>(0.34; 0.51)	0.56 ± 0.029	0.54<br>(0.51; 0.64)	Student's<br>t-test	0.018	1.14
Ki number/0.01 mm^2 of the gland surface	6.6 ± 0.73	6.8<br>(6.1; 7.3)	10.1 ± 1.14	9.6<br>(7.6; 12.6)	Student's<br>t-test	0.028	1.36
**Figure 4.** Immunohistochemical reactions for Ki-67 and cadherin in the sections of the jejunum							
Intensity of expression of cadherin in the jejunum, mean pixel intensity	171 ± 3.3	172<br>(169; 176)	143 ± 7.4	142<br>(133; 156)	Welch's t-test	<0.001	1.87
**Table 2.** The histomorphometrical parameters of the enteric nervous plexuses in the duodenum and jejunum							
Auerbach plexus, duodenum							
Area, µm^2	828 ± 58.4	784<br>(726; 951)	656 ± 63.3	628<br>(519; 748)	Student's<br>t-test	0.074	
Perimeter, µm	137 ± 27.3	140<br>(123; 167)	131 ± 15.1	130<br>(94; 156)	Mann-<br>Whitney test	0.689	
Mean Feret diameter, µm	41.0 ± 13.63	28.1<br>(21.9; 48.7)	39.3 ± 16.70	22.2<br>(8.7; 84.2)	Mann-<br>Whitney test	0.471	
Mean diameter, µm	25.4 ± 4.16	28.5<br>(23.5; 30.4)	20.3 ± 3.80	23.4<br>(13.9; 25.2)	Student's<br>t-test	0.387	
Min diameter, µm	15.9 ± 1.88	15.9<br>(13.2; 16.7)	11.8 ± 1.47	12.6<br>(7.7; 14.7)	Student's<br>t-test	0.116	
Sphericity	0.31 ± 0.077	0.23<br>(0.16; 0.46)	0.30 ± 0.090	0.22<br>(0.17; 0.56)	Student's<br>t-test	0.934	
The number of the ganglia, /mm	5.5 ± 0.37	5.3<br>(5.0; 6.5)	5.6 ± 0.49	5.5<br>(4.5; 6.0)	Student's<br>t-test	0.874	

Meissner plexus, duodenum							
Area, µm^2	384 ± 46.5	403 (287; 467)	372 ± 58.0	349 (316; 512)	Student's t-test	0.875	
Perimeter, µm	82 ± 10.2	78 (60; 105)	80 ± 13.1	91 (48; 105)	Student's t-test	0.906	
Mean Feret diameter, µm	24.6 ± 3.02	25.7 (16.7; 31.5)	24.5 ± 3.87	25.3 (17.4; 28.4)	Mann-Whitney test	0.936	
Mean diameter, µm	20.8 ± 2.57	21.8 (18.8; 25.8)	20.1 ± 2.53	18.4 (17.2; 22.4)	Student's t-test	0.851	
Min diameter, µm	14.6 ± 2.00	14.2 (11.2; 19.5)	14.2 ± 1.80	14.9 (12.4; 16.6)	Student's t-test	0.885	
Sphericity	0.29 ± 0.077	0.28 (0.16; 0.46)	0.31 ± 0.020	0.31 (0.29; 0.36)	Welch's t-test	0.812	
The number of the ganglia, /mm	1.5 ± 0.08	1.5 (1.3; 1.6)	4.4 ± 0.33	4.3 (4.0; 4.6)	Student's t-test	<0.001	4.54
Auerbach plexus, jejunum							
Area, µm^2	585 ± 51.0	564 (487; 675)	979 ± 60.8	1022 (820; 111)	Student's t-test	<0.001	2.62
Perimeter, µm	120 ± 22.5	105 (78; 176)	179 ± 30.2	194 (152; 236)	Mann-Whitney test	0.173	
Mean Feret diameter, µm	36.2 ± 3.88	36.2 (28.1; 41.9)	53.6 ± 4.94	54.7 (46.8; 61.4)	Student's t-test	0.020	1.46
Mean diameter, µm	12.9 ± 2.65	12.7 (7.9; 19.3)	13.0 ± 2.08	11.3 (8.6; 18.4)	Student's t-test	0.977	
Min diameter, µm	21.9 ± 4.12	19.8 (14.0; 28.2)	24.7 ± 5.02	30.1 (14.1; 34.1)	Student's t-test	0.676	
Sphericity	0.21 ± 0.077	0.17 (0.05; 0.31)	0.13 ± 0.045	0.12 (0.04; 0.15)	Mann-Whitney test	0.472	
The number of the ganglia, /mm	3.0 ± 0.45	3.0 (2.1; 3.6)	3.1 ± 0.24	3.2 (3.0; 3.5)	Welch's t-test	0.850	
Meissner plexus, jejunum							

Area, μm^2	355 ± 42.0	385 (246; 445)	375 ± 64.5	397 (297; 480)	Student's t-test	0.830	
Perimeter, μm	80 ± 9.0	81 (63; 97)	79 ± 13.9	90 (43; 95)	Student's t-test	0.953	
Mean Feret diameter, μm	24.0 ± 2.74	25.9 (20.0; 29.3)	23.9 ± 4.16	24.5 (15.4; 32.1)	Student's t-test	0.984	
Mean diameter, μm	19.3 ± 2.29	17.6 (15.6; 20.3)	19.4 ± 2.94	20.7 (11.3; 24.1)	Mann-Whitney test	0.810	
Min diameter, μm	12.8 ± 1.84	12.4 (8.79; 16.7)	13.7 ± 2.16	13.5 (12.3; 14.8)	Student's t-test	0.758	
Sphericity	0.31 ± 0.073	0.28 (0.20; 0.46)	0.33 ± 0.073	0.30 (0.13; 0.49)	Student's t-test	0.874	
The number of the ganglia, /mm	3.8 ± 0.37	3.7 (3.1; 4.7)	3.9 ± 0.04	3.9 (3.8; 3.9)	Welch's t-test	0.797	
**Table 3.** The histomorphometrical parameters of the liver							
Total cell number, /mm^2	2084 ± 77.2	2038 (1985; 2152)	2647 ± 56.3	2645 (2546; 2721)	Student's t-test	<0.001	3.22
Total hepatocyte number, /mm^2	1565 ± 30.6	1561 (1506; 1624)	2149 ± 22.5	2150 (2100; 2176)	Student's t-test	<0.001	8.11
Total hepatocyte nucleus numer, /mm^2	1651 ± 26.5	1652 (1628; 1664)	2257 ± 25.3	2246 (2201; 2302)	Student's t-test	<0.001	8.71
Mononuclear hepatocytes nucleus number, /mm^2	1479 ± 48.6	1457 (1386; 1607)	2041 ± 64.1	2026 (1942; 2071)	Student's t-test	<0.001	3.68
Binuclear hepatocytes nucleus number, /mm^2	86 ± 11.4	90 (65; 105)	108 ± 11.8	109 (97; 128)	Student's t-test	0.211	
Non-hepatocyte cells number, /mm^2	519 ± 40.4	552 (499; 588)	488 ± 20.8	460 (452; 538)	Mann-Whitney test	0.230	
Immature collagen, %	15.8 ± 1.84	16.2 (11.9; 19.7)	39.8 ± 2.16	41.0 (37.1; 42.9)	Student's t-test	<0.001	4.46
**Figure 5.** Immunohistochemical reactions for nesfatin-1 in the jejunal crypts and Auerbach plexus							
The intensity of expression of nesfatin-1 in the jejunum, mean pixel intensity	199 ± 2.9	200 (190; 204)	246 ± 1.6	248 (242; 249)	Student's t-test	<0.001	7.44
**Figure 5.** Immunohistochemical reactions for ZO-1 in jejunum and duodenum							

The intensity of expression of ZO-1 in the jejunum, mean pixel intensity	167 ± 3.7	171 (155; 174)	129 ± 3.8	126 (122; 136)	Mann-Whitney test	<0.001	4.00
The intensity of expression of ZO-1 in the duodenum, mean pixel intensity	171 ± 3.3	172 (168; 178)	143 ± 3.5	146 (137; 147)	Student's t-test	<0.001	3.20
The intensity of expression of ZO-1 in the duodenal villi, mean pixel intensity	158 ± 3.7	157 (154; 167)	135 ± 3.3	134 (129; 136)	Student's t-test	<0.001	2.45
**Figure 7.** Immunohistochemical reactions for occludin in jejunum and duodenum							
The intensity of expression of occludin in the jejunum, mean pixel intensity	133 ± 4.5	133 (127; 140)	159 ± 5.7	161 (150; 167)	Student's t-test	0.004	1.89
The intensity of expression of occludin in the duodenum, mean pixel intensity	167 ± 4.2	168 (159; 174)	171 ± 5.1	174 (161; 180)	Student's t-test	0.558	
The intensity of expression of occludin in the duodenal villi, mean pixel intensity	149 ± 6.6	150 (135; 162)	110 ± 7.5	105 (96; 114)	Student's t-test	<0.001	2.06
**Figure 8.** Immunohistochemical reactions for marvelD3 in jejunum and duodenum							
The intensity of expression of marvelD3 in the jejunum, mean pixel intensity	182 ± 5.5	183 (180; 190)	185 ± 6.3	183 (178; 200)	Student's t-test	0.726	
The intensity of expression of marvelD3 in the duodenum, mean pixel intensity	173 ± 3.0	176 (170; 177)	189 ± 2.7	189 (185; 194)	Student's t-test	0.003	2.09
The intensity of expression of marvelD3 in the duodenal villi, mean pixel intensity	158 ± 6.5	163 (158; 167)	137 ± 3.2	139 (134; 143)	Mann-Whitney test	0.045	0.58
**Figure 9.** Immunohistochemical reactions for ghrelin in jejunum and duodenum							
The intensity of expression of ghrelin in the jejunum, mean pixel intensity	152 ± 4.2	150 (142; 164)	176 ± 2.9	177 (169; 182)	Student's t-test	<0.001	2.50
The intensity of expression of ghrelin in the duodenum, mean pixel intensity	151 ± 5.1	153 (143; 161)	147 ± 2.1	146 (143; 150)	Welch's t-test	0.496	
The intensity of expression of ghrelin in the duodenal Auerbach, mean pixel intensity	164 ± 4.8	166 (161; 172)	212 ± 9.2	216 (202; 231)	Student's t-test	<0.001	2.39
**Figure 10.** Immunohistochemical reactions for leptin in jejunum and duodenum							
The intensity of expression of ghrelin in the jejunum, mean pixel intensity	161 ± 5.1	163 (156; 171)	165 ± 6.6	163 (152; 176)	Student's t-test	0.649	
The intensity of expression of ghrelin in the jejunal crypts, mean pixel intensity	164 ± 2.5	162 (160; 169)	167 ± 6.0	165 (154; 179)	Welch's t-test	0.745	

The intensity of expression of ghrelin in the duodenum, mean pixel intensity	140 ± 3.3	138 (137; 142)	167 ± 4.7	165 (156; 178)	Student's t-test	<0.001	2.42
The intensity of expression of ghrelin in the duodenal Auerbach, mean pixel intensity	159 ± 7.9	159 (152; 167)	198 ± 2.5	200 (191; 202)	Welch's t-test	<0.001	2.43
**Figure 11.** Immunohistochemical reactions for VIP in Auerbach plexuses of duodenum and jejunum							
The intensity of expression of ghrelin in the duodenal Auerbach, mean pixel intensity	135 ± 12.3	145 (115; 149)	178 ± 4.4	180 (167; 184)	Welch's t-test	0.004	1.73
The intensity of expression of ghrelin in the jejunal Auerbach, mean pixel intensity	156 ± 4.9	163 (156; 170)	189 ± 1.3	189 (187; 190)	Student's t-test	0.001	2.40
